# Carbohydrate Mouth Rinse and Spray Improve Prolonged Exercise Performance in Recreationally Trained Male College Students

**DOI:** 10.3390/sports10040051

**Published:** 2022-03-29

**Authors:** Asako Shirai, Tsuyoshi Wadazumi, Yoko Hirata, Naomi Hamada, Nobuko Hongu

**Affiliations:** 1 Department of Sport Education, Osaka University of Health and Sport Sciences, 1-1 Asashirodai, Kumatori-cho, Sennan-gun 590-0496, Osaka, Japan; 2 Faculty of Health and Well-Being, Kansai University, 1-11-1, Kaorigaoka-cho, Sakai-ku, Sakai 590-8515, Osaka, Japan; wadazumi@kansai-u.ac.jp (T.W.); yo-hirata@kwjc.kobe-wu.ac.jp (Y.H.); hamada-n@higashiosaka.ac.jp (N.H.); 3 Department of Food and Nutritional Science, Kobe Women’s Junior College, 4-7-2 Nakamachi, Minatojima, Chuo-ku, Kobe 650-0046, Hyogo, Japan; 4 Department of Applied Food Science, Higashiosaka Junior College, 3-1-1, Nishizutsumigakuen-cho, Higashiosaka 577-8567, Osaka, Japan; 5 Department of Food and Human Life Science, Osaka Metropolitan University, 3-3-138, Sugimoto, Sumiyoshi-ku 558-8585, Osaka, Japan; kay.hongu@gmail.com

**Keywords:** carbohydrate mouth rinse, mouth spray, exercise performance, nutrition, exercise fatigue, central fatigue

## Abstract

Mouth rinsing with a carbohydrate (CHO) solution has emerged as a sports nutrition strategy to increase endurance performance. This study aimed to clarify the effects of two forms of CHO sensing in the mouth (i.e., CHO mouth rinse (CMR) and CHO mouth spray (CMS)) on exercise performance during prolonged exercise, including ultra-high intensity intermittent exercise over time. We conducted the following experimental trials: (1) 6% glucose solution (G), (2) 6% CMR, (3) 6% CMS, and (4) water (WAT). These trials were conducted at least 1 week apart in a randomized crossover design. Eight male college students performed constant-load exercise for 60 min (intensity 40% VO_2_peak), four sets of the Wingate test (three 30 s Wingate tests with a 4 min recovery between each test), and a constant-load exercise for 30 min (intensity 40% VO_2_peak). The mean exercise power output (Watt), ratings of perceived exertion, and blood glucose levels were measured. We found that the mean power values of the CMR and CMS in the third and fourth sets was significantly higher than that of WAT (*p* < 0.05), and that the G trial did not show a significant difference from any other trial. Thus, when compared to G or WAT, CMR and CMS can help improve endurance exercise performance.

## 1. Introduction

During prolonged exercise (defined as exercise lasting for more than 2 h), such as marathons, trail runs, and triathlons, substantial amounts of muscle glycogen are consumed. As glycogen depletion leads to poor exercise performance, it is beneficial to increase the intake of carbohydrates (CHO) before and during prolonged exercise. Ingested CHOs are metabolized via pathways that preserve muscle glycogen and maintain blood glucose levels [1]. Recently, CHO mouth rinses, which do not involve ingesting the CHO, have been introduced to maintain performance during exercises lasting for 30–75 min or during high-intensity exercise when there is no significant reduction in blood glucose levels [2,3,4,5].

To use the CHO mouth rinse, the oral cavity is first rinsed with a low-concentration CHO solution for 5–10 s, which is then spit out without ingesting. It is speculated that the CHO mouth rinse effect involves a neural mechanism mediated by taste buds expressing CHO receptors in the oral cavity [6]. The CHO mouth rinse activates brain regions involved in the reward system, including the dorsolateral prefrontal cortex, striatum, and anterior cingulate cortex; however, no hematological or biochemical effects have been reported [4,5,7].

To date, extensive studies have been conducted on the ergogenic effects of CHO mouth rinse, including those assessing its effects on endurance exercise lasting approximately 1 h [4,5,8,9,10,11], exercise with isometric maximal voluntary contraction [12,13], and high-intensity intermittent exercise [14,15]. Recently, Karayigit et al. (2022) reported that mouth rinsing with a 6% CHO solution for three times enhances 80% of 1 repetition maximum (RM) bench press repetitions to failure in resistance-trained males [16]. These results suggest that the CHO mouth rinse has beneficial effects on high-intensity intermittent exercise, especially at the later stage of the exercise [17], and that it attenuates the decrease in performance power in conditions associated with locomotor fatigue [13]. However, the effect of the CHO mouth rinse during sprint performance remains controversial [18] and may depend on the nutritional states (e.g., feeding or fasting) of individuals before performance [7,8,19]. However, several issues require clarification prior to the use of the CHO mouth rinse in actual sporting situations. For example, its effect under prolonged exercise conditions (>2 h) has not been clarified. In addition, the potential for CHO supplementation to cause gastrointestinal discomfort during exercise and the fact that CHO is not used as an energy substrate immediately after ingestion remain undefined. Gam et al. noted that repeated rinsing of the mouth during exercise may restrict breathing and affect its rhythm, thereby negatively affecting performance [20]. The need to spit out the CHO solution after mouth rinsing also creates a hygiene issue, thus limiting its use.

This study aimed to investigate whether CHO mouth rinse, CHO mouth spray, or CHO solution ingestion affects performance during prolonged exercise, including ultra-high intensity intermittent exercise. We hypothesized that the CHO mouth spray and CHO mouth rinsing would enhance endurance exercise performance and high-intensity intermittent exercise [4,5,7,8,9,10,11,12,13,14,15,16,18,19].

## 2. Materials and Methods

### 2.1. Participants

The study participants were healthy male university students. The physical characteristics of the participants are listed in Table 1. They were fully informed about the purpose, methods, and possible risks of this study and voluntarily signed a consent form. The participants were instructed to maintain a normal diet and physical activity for the duration of the study.

They were instructed not to engage in any strenuous physical activity, consume caffeine, or drink alcohol during the 24 h period prior to each test; they were also advised to fast from 21:00 the day before the test. All testing sessions were conducted in the morning.

### 2.2. Preliminary Test

At least 1 week before the start of the experimental trials, the VO_2_peak of each participant was determined using an electromagnetic brake-type bicycle ergometer (AEROBIKE 75XLIII, Konami Holdings Corporation, Tokyo, Japan) and an expired gas analyzer (AEROMONITOR AE-310S, Minato Medical Science Co., Ltd., Osaka, Japan). Following a 2 min warm-up at 20 W, the workload was increased by 20 W every min until volitional exhaustion. During the test, ventilation and gas exchanges were measured. The 40% VO_2_peak was determined based on the detected values. Before the experiment, the participants undertook some trial runs with the Wingate test (POWERMAX-V III, Konami Holdings Corporation, Tokyo, Japan) for familiarization.

### 2.3. Experimental Design

At 9:00 am, the participants assembled in a laboratory maintained at 25 °C. They performed four experimental trials following the use of 6% CHO (glucose) solution (G), 6% CHO mouth rinse (CMR), 6% CHO mouth spray (CMS), or water (WAT). Participants were randomly assigned to the order of the four trials using computer-generated random numbers (randomized crossover design), and each trial was performed at least 1 week apart.

#### 2.3.1. Wadazumi Protocol

**Figure 1:**
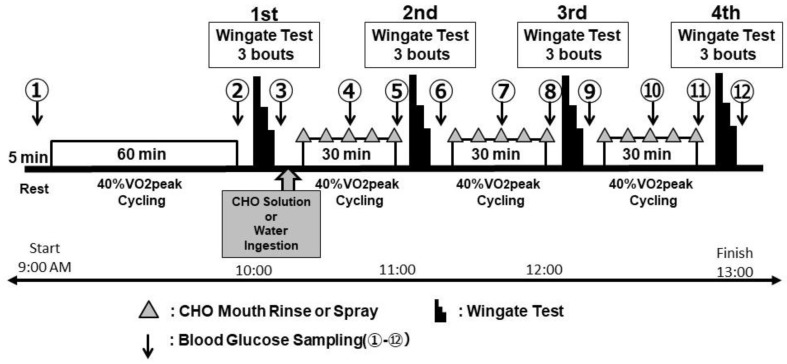
Experimental design of the Wadazumi protocol. The cycling exercise protocol used in this study combined a constant-load exercise with a Wingate test. The strength of the constant load was set to 60 min and 30 min for three sets ×40% VO_2_peak. In total, four sets of Wingate tests (30 s × 3) were performed. After the first set of Wingate test, participants consumed the CHO solution in the G trial and water in the WAT trial. In the CMR and CMS trials, mouth rinse or spray were used at every 7.5 min intervals. Points (①–⑫) indicate the timepoints when blood glucose levels and RPE were assessed.

**Figure 2:**
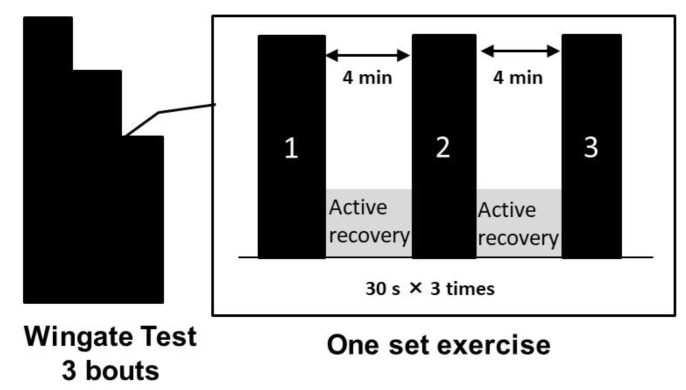
Wingate Test.

The cycling exercise protocol used in this study was a combination of constant-load exercise and the Wingate test (Figure 1 and Figure 2) [21,22]. The participants performed a constant-load exercise for 60 min using an electromagnetic brake-type bicycle ergometer at 40% VO_2_peak intensity, and a Wingate test with four sets combined with a constant-load exercise at 40% VO_2_peak intensity for 30 min.

Following the first set of the Wingate test, participants in the G trials ingested 500 mL of the CHO solution. In the CMR trials, participants used CHO mouth rinse during the three sets of constant-load exercise. In the CMS trials, participants used the CHO mouth spray during the three sets of constant-load exercise. In the WAT trials, participants ingested 500 mL of water. Participants were free to drink water in all trials, and their water intake was recorded.

#### 2.3.2. Wingate Test

The Wingate test, which was performed on an electromagnetic brake-type bicycle ergometer, was used as an indicator of exercise performance. The exercise mode is shown in Figure 2. A load of 0.075 kg per kg body weight of the participant was applied to the pedal. The Wingate test was performed for 30 s thrice, with a 4 min active recovery rest period between each test, in which the participants pedaled at their own pace against unloaded resistance. One Wingate test set included three individual Wingate tests. The investigator instructed the participants to maintain their cycling at full power in all sets. The participants were constantly encouraged during the tests to enable them to give their best performance during the three bouts of the Wingate test.

### 2.4. Carbohybrate Solution, Mouth Rinse and Mouth Spray

**Figure 3:**
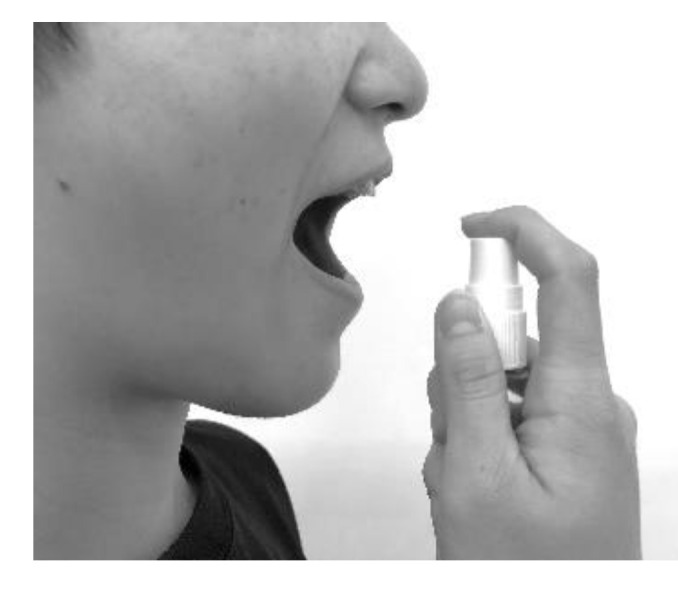
Mouth Spray.

A 6% glucose (Wako Pure Chemical Industries, Osaka, Japan) solution was used as the CHO drinking solution, mouth rinse, and mouth spray for the experiments. Participants rinsed with 25 mL of the mouth rinse solution for 10 s and spit it out into another container without swallowing. The mouth sprays were designed such that the same amount of CHO solution as in the mouth rinse stayed in the oral cavity. As a pre-test, the participants were asked to rinse 25 mL CHO solution for 10 s without swallowing. An average reduction of 0.54 mL in the amount spat was noted. Thus, 0.54 mL would be used for oral exposure. Therefore, the oral cavity was sprayed six times using a container that can spray 0.1 mL of CHO solution with one push (Figure 3). The participants were instructed on the right way to use the mouth spray and to be careful not to swallow the solution during the process.

### 2.5. Assessment of Exercise Performance

#### 2.5.1. Exercise Performance

In the Wingate test, the “average power value” for 30 s and the “maximum power value” in which the power value per 5 s reached the maximum value were evaluated. This study used the “average power value” as an index of exercise performance.

#### 2.5.2. Ratings of Perceived Exertion (RPE)

RPE were measured using the Borg scale [23] and simultaneously assessed with blood glucose levels.

#### 2.5.3. Blood Glucose Levels

Blood samples were taken from the fingertip, and blood glucose levels were assessed from the resting state (immediately before the test) to the end of tests ① to ⑫ (Figure 1) using a glucometer (Glutest Neo Super; Sanwa Kagaku Kenkyusho, Nagoya, Japan).

### 2.6. Statistical Analysis

Normality was confirmed using the Shapiro–Wilk test. Thereafter, exercise performance was calculated using the first average power value (in W) of the first set of the Wingate tests as 100%; the mean of three power values was calculated.

To compare the effects of each trial result based on the solution consumed, the test of sphericity of Mauchly was performed by one-way, repeated-measure analysis of variance (ANOVA). If sphericity could not be presumed, the degrees of freedom were corrected using the Huynh–Feldt epsilon. Subsequently, Bonferroni multiple comparison test was performed. SPSS Statistics ver. 27.0 (IBM Corporation) was used for all statistical analyses. All measured values are expressed as the mean ± standard deviation (mean ± SD). Statistical significance was set at *p* < 0.05.

### 2.7. Ethics

This study was conducted in accordance with the tenets of the Declaration of Helsinki. Ethical approval was obtained from the Ethics Committee of the Faculty of Human Health, Kansai University (approval number 2020-1, approved on 6 August 2020).

## 3. Results

### 3.1. Average Power Value for Each Set of the Wingate Tests

**Figure 4:**
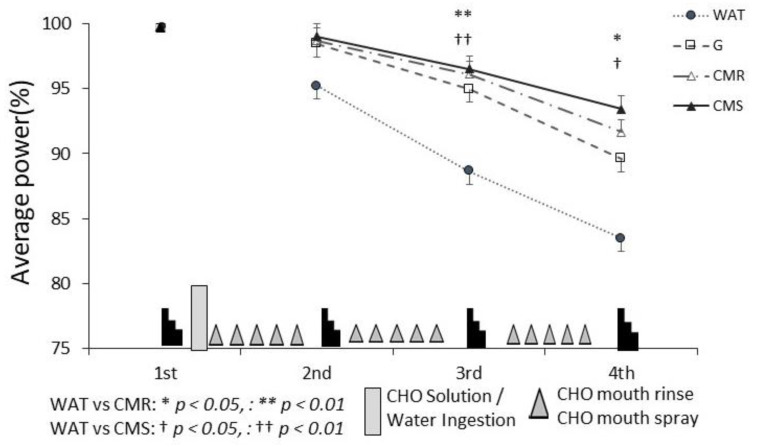
Average power assessments. Comparison of the average power values in the Wingate test (2–4 sets) in each trial. For each trial, the values of the Wingate test performance in the second to fourth sets were compared with and expressed as percentages of those in the first set (*N* = 8). WAT, water; G, CHO Solution; CMR, CHO mouth rinse; CMS, CHO mouth spray.

Figure 4 shows the results of the comparison of the average power values among the exercise sets for each trial. When compared to the average power value of the first set of Wingate tests (100%), the average power value gradually decreased across sets 2–4 in all four trials. One-way ANOVA revealed a main effect in the third (F(3,21) = 6.546, *p* = 0.03) and fourth sets (F(1.684,11.787) = 4.430, *p* = 0.042). Post hoc tests showed that the average power values for the third set were as follows: WAT (88.65 ± 2.6%), G (94.9 ± 4.0%), CMR (96.12 ± 3.8%), and CMS (96.5 ± 3.2%). The results were significantly higher for CMR and CMS than for WAT (WAT vs. G, *p* = 0.147, WAT vs. CMR, *p* = 0.007, WAT vs. CMS, *p* = 0.002). In the fourth set, the average power values were as follows: WAT (83.5 ± 4.0%), G (89.6 ± 6.8%), CMR (91.6 ± 5.4%), CMS (93.4 ± 5.3%); CMR and CMS had significantly higher values than WAT (WAT vs. G, *p* = 0.314, WAT vs. CMR, *p* = 0.021, WAT vs. CMS, *p* = 0.013). One-way ANOVA of the mean power values for CMR and CMS also revealed no main effects in every set (CMR vs. CMS, non-significant).

### 3.2. RPE

**Figure 5:**
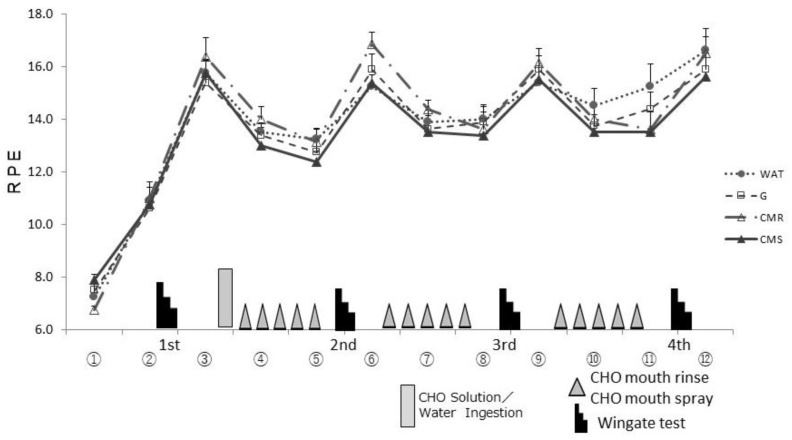
Changes in the Ratings of Perceived Exertion (RPE) in each trial. WAT, water; G, CHO Solution; CMR, CHO mouth rinse; CMS, CHO mouth spray. The 12 points (①–⑫) indicate the timepoints when RPE were assessed.

The RPE increased after each set of the Wingate test (③, ⑥, ⑨, ⑫) in all four trials. However, there was no significant difference between the trials (Figure 5).

### 3.3. Blood Glucose Levels

**Figure 6:**
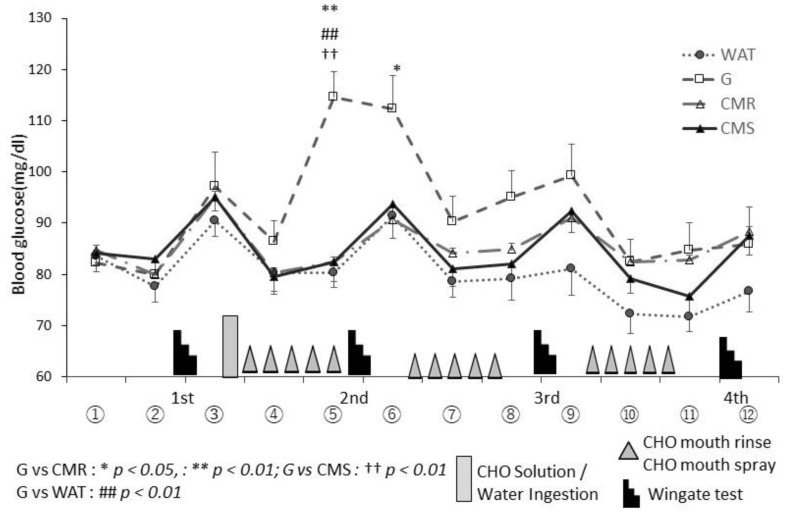
Changes in blood glucose in each trial. WAT—water; G—CHO Solution; CMR—CHO mouth rinse, CMS—CHO mouth spray. The 12 points (①–⑫) indicate blood glucose sampling.

The changes in blood glucose levels during the three trials are shown in Figure 6. In all four trials, blood glucose levels increased after the first set of the Wingate test. Thirty minutes after consumption of the CHO solution (⑤), participants in the G trial (114.6 ± 14.8 mg/dl) had a sharp increase in blood glucose levels (F(3,21) = 22.647, *p* < 0.01), (G vs. WAT, CMR, CMS, *p* < 0.01). Following the second set of the Wingate test (⑥), those in the WAT, CMR, and CMS trials showed a slight increase in blood glucose levels (F(3,21) = 8.578, *p* < 0.01), (G vs. WAT, *p* = 0.137, G vs. CMR, *p* = 0.021, G vs. CMS, *p* = 0.71). Following the third set of the Wingate test (⑨), those in the G, CMR, and CMS trials had a slight increase in blood glucose levels, which was not significant (F(3,21) = 3.112, *p* = 0.48). No significant differences in blood glucose levels were noted following the fourth set of Wingate tests (⑫) (F(3,21) = 1.142, *p* = 0.246).

## 4. Discussion

The effects of CHO ingestion, CMR, CMS, and WAT ingestion on exercise performance were investigated using the Wadazumi protocol, which is a mixture of ultra-high-intensity intermittent and endurance exercise. We found that the G trial had a higher average power value than the WAT trial, although no significant difference was observed. Moreover, CMR and CMS trials attenuated the decrease in exercise power during the later stage of the ultra-high-intensity intermittent exercise compared with the WAT trial. The use of CMR and CMS did not significantly change blood glucose levels during exercise, and there was no difference in the effects of the two forms of CHO mouth rinsing on improving exercise performance.

### 4.1. Effects of Mouth Rinse on Ultra-High Intensity Intermittent Exercise

We found a significant difference in the performance between the CMR and WAT trials, as well as between the CMS and WAT trials in the third and fourth sets at the end of the exercise, which exceeded 3 h from the start of ultra-high-intensity intermittent exercise; however, the performance in the G trial (6% CHO solution) was not significantly different from that in the WAT trial. In addition, the effect of the CMR trial was not different from that in the CMS trial. A previous study [21] that employed the same research protocol as this study reported a significant attenuation in performance decline in participants who ingested an 8% CHO solution compared with those who drank only water. Thus, we speculate that the single ingestion of the 6% CHO solution used in this study resulted in a decreased performance owing to energy depletion.

CHO mouth rinses have been reported to improve ultra-high-intensity intermittent exercise performance over 45 min [14,15]. Rollo et al. reported no significant effect 60 min after the start of exercise, but an improvement in performance during the final set of 75–90 min was observed [15]. This effect was attributed to the decrease in glycogen storage due to prolonged exercise. In contrast, Dorling and Earnest found no effect after 60 min of high-intensity exercise [24]. In their study, the degree of exercise fatigue was low, and the glycogen storage may not have been depleted.

Blood glucose levels in the G trial were significantly higher than those in other trials; no changes in blood glucose were observed in the WAT, CMR, and CMR trials (Figure 6). This finding is consistent with that of a previous study [4]. The total amount of energy attributed to the use of the mouth rinse or mouth spray was approximately 2.2 kcal (6% CHO solution: 0.6 mL × 15). In this study, a mixture of steady load and ultra-high intensity intermittent exercise over a period more than 4 h showed that the average power in CMR and CMS trials was maintained until the later stage of the exercise, despite the depletion of endogenous energy. Therefore, the mechanism underlying the maintenance of exercise performance is different from that underlying energy intake [5,12,25].

### 4.2. Relationship between CHO Mouth Rinse and Motor Fatigue

Exercise fatigue is defined as a state in which a person cannot exert the necessary force for exercise [26]; prolonged exercise leads to the depletion of glycogen in the muscles and liver, which subsequently results in central fatigue [27]. In addition, information about factors of fatigue that occur during long hours of exercise, such as lack of concentration and motivation [28], is integrated into the brain, creating “central fatigue” [29]. These signals are consciously or unconsciously fed forward from the brain to exercise, causing a decline in exercise performance [28].

The effects of CHO mouth rinses on the attenuation of a decrease in prolonged exercise performance is speculated to involve neural mechanisms [6]. In response to oral CHO, brain regions within the dorsolateral prefrontal cortex, anterior cingulate cortex, and caudate nucleus, which form part of the striatum, are activated [4]; the orbitofrontal cortex, which is part of the reward value of taste, is also involved [30,31]. Furthermore, CHO mouth rinses increase the executive function and self-control in the brain [32,33,34]. Central depletion, including self-control, enhances the ergogenic effect of CHO mouth rinses [32].

Other studies have shown that a CHO mouth rinse improves performance and RPE during resistance exercise with peripheral fatigue [35], even in the absence of central fatigue [12,13,36]. In addition, the fasting state is more effective than the feeding state, indicating that the fatigue state and the effect of CHO mouth rinse may be enhanced when the central fatigue state or energy is depleted.

In our study, significant improvements in exercise performance were observed in the third and fourth sets. Towards the end of prolonged intermittent exercise, which is associated with muscle and liver glycogen depletion, the CHO mouth rinse and CHO mouth spray may have activated brain regions involved in reward and motor control, resulting in improved performance.

### 4.3. Prospects for CHO Mouth Spray

To date, the use of CHO mouth rinse has been extensively studied by basic research in the laboratory. The results of our study revealed the potential for CHO mouth rinses in sports competitions in which the difference in records is less than 1 s. However, many issues remain to be addressed for its application in actual sports settings, most notably, the act of spitting the solution after mouth rinsing may not be suitable during play in the track field, a gymnasium, or a stadium.

Mouth spray formulations provide an easy and hygienic delivery system that can be used just before or during competitions. The spray-type mouth rinse used in this study is a simple and hygienic method of delivery that can contribute to the development of basic research on the new possibilities of the effects of CHO mouth rinse. Further research is needed in practice for such nutritional strategies.

### 4.4. Limitations

This study had some limitations. First, eight participants were included in the four trials. Although the number of participants in this study is small, this study was a valuable one in which the protocol was conducted for more than 4 h under the same conditions. Second, hematological and other biochemical parameters could not be investigated. Third, it is necessary to consider each person’s dietary management and individual differences in fatigue tolerance. In the future, we will consider using a larger sample size to incorporate this strategy for improving prolonged exercise performance.

## 5. Conclusions

During ultra-high intensity intermittent and endurance exercise sessions, CMR and CMS were found to be significantly more effective than G or WAT in reducing the decline in performance in the exercise fatigue state.

Both CMR and CMS may be useful in various sports situations. Further studies are required to verify and further develop the appropriate use of CMR and CMS.

## Figures and Tables

**Table 1:** Physical characteristics of the participants (*N* = 8).

	Age (Years)	Height (cm)	Weight (kg)	Fat (%)	40% VO_2_peak (mL/min)	Load (40% VO_2_peak) (W)
Mean	22.3	171.9	67.0	21.1	1212.0	103.2
SD	1.3	4.7	6.63	3.15	112.5	12.69

## Data Availability

The data presented in this study are available on request from the corresponding author and the permission of all parties involved in the study.
